# Polymorphism on Chromosome 9p21.3 Is Associated with Severity and Early-Onset CAD in Type 2 Diabetic Tunisian Population

**DOI:** 10.1155/2015/792679

**Published:** 2015-08-31

**Authors:** Kaouthar Abid, Donia Mili, Abderraouf Kenani

**Affiliations:** Laboratory of Biochemistry, UR 12ES08, Faculty of Medicine, 5000 Monastir, Tunisia

## Abstract

Multiple association studies found that the human 9p21.3 chromosome locus is a risk factor for atherosclerosis. The purpose of this study was to investigate the association of the severity and early-onset of coronary artery disease with variant rs1333049 on chromosome 9p21.3 polymorphism and the impact of this variant on cardiovascular risk factors in type 2 diabetic patients. The study population consisted of a control CAD group (101 patients) and 273 consecutive type 2 diabetic patients. Severity and extent of coronary atherosclerosis were scored numerically using the Gensini scoring system. The diabetic population was divided into three groups according to Gensini score: Group 1: no stenosis; Group 2: moderate CAD; Group 3, severe CAD. The homozygous CC genotype of rs1333049 was significantly associated with CAD in Group 2 (OR: 1.36; *p* = 0.02) and Group 3 (OR: 5.77, *p* < 0.001) compared to Group 1 (OR: 0.18; *p* = 0.2) and control group (OR: 0.22; *p* = 0.21). Among diabetic patients with early-onset CAD, CC genotype carriers had significantly higher Gensini scores than non-CC genotype carriers (49 ± 21.3 versus 14.87 ± 25.22; *p* < 0.001). The homozygous CC genotype of rs1333049 confers a magnified risk of early-onset and severe CAD in type 2 diabetic Tunisian population.

## 1. Introduction

The association of genetics and environmental factors (such as life-style) plays a major role in the pathogenesis of coronary artery disease (CAD) and type 2 diabetes (T2D). The genetic architecture of CAD and that of T2D are dependent on multiple genetic and environmental triggers that may be specific to each disease. The chromosome 9p21.3 has been identified as the locus with strongest association with coronary artery disease in multiple independent large-scale genome-wide association studies [1–3].

It is the most consistently replicated genetic locus for CAD [1, 4] and T2D [5, 6]. Recent genome-wide scanning has implicated chromosome 9p21.3 as a locus conferring susceptibility to CAD [2, 7–9]. This increased risk is independent of traditional risk factors, including gender, age, obesity, smoking, hypertension, and hyperlipidemia [5]. Since rs1333049 polymorphism on chromosome 9p21.3 has a strong association with CAD [10], we hypothesized that such SNP on chromosome 9p21.3 may have an amplifying impact on early-onset and severity of CAD. This is the first study evaluating the association between 9p21.3 and CAD in Tunisian population. The purpose of the present study was to investigate the relationship between one SNP (single nucleotide polymorphism), rs1333049, on chromosome 9p21.3 and susceptibility to CAD, the effect of this SNP on cardiovascular risk factors, severity, and early-onset of CAD in type 2 diabetic Tunisian population.

## 2. Methods

### 2.1. Patients

The study population consisted of a total number of 374 patients composed of a control CAD group of 101 nondiabetic angiographic control cohort with CAD and 273 consecutive type 2 diabetic patients undergoing a routine coronary angiography to evaluate suspected CAD. Subjects were defined with coronary artery disease (CAD) when presenting a stenosis >50% in at least one major coronary artery. Subjects were defined without coronary artery disease (no CAD) when presenting a stenosis <50 in at least one major coronary artery. The severity and the extent of coronary artery disease were evaluated with the Gensini score [11]. The Gensini score was computed by assigning a severity score to each coronary stenosis according to the degree of luminal narrowing and its geographic importance. The study population was divided into three groups: Group 1: patients with no stenosis (Gensini score = 0); Group 2: patients with moderate CAD (Gensini score < 40); Group 3: patients with severe CAD (Gensini score > 40). Hypertension was diagnosed as blood pressure of higher than 140/90 mmHg, which was measured according to guidelines [12] and/or the current use of antihypertensive drugs. Diabetic subjects were defined by a fasting plasma glucose >7.0 mmol/L or by the use of antidiabetic drugs [13]. Hyperlipidemia was defined by either high cholesterol levels (TC > 5.17 mmol/L) or high triglycerides levels (TG > 1.70 mmol/L) or both or by the use of hypocholesterolemic drugs. Obese subjects were defined by a BMI >30.0 Kg/m^2^. Data on age, sex, smoking, and smoking history were collected from the participants' medical records or by direct interviews. Fasting concentrations of TC, TG, LDL-C, and HDL-C were measured using standard methods [14]. Patients were diagnosed with hyperlipidemia if they had serum levels of total cholesterol (TC) >5.7 mmol/L, triglycerides (TG) >1.7 mmol/L, low-density lipoprotein cholesterol (LDL-C) >3.64 mmol/L, or high-density lipoprotein cholesterol (HDL-C) <0.91 mmol/L. Early-onset CAD was defined as clinical CAD occurring by age ≤55 years in male or ≤60 years in female patients [15, 16]. This study was approved by our hospital ethical committee. All participants were of Tunisian origin and gave their informed consent for this study.

### 2.2. Biochemical Analysis

Blood samples were taken for biochemical analysis following overnight fasting. CT, TG, and HDL-C concentrations were determined at accredited clinical laboratories using routine clinical methods. LDL-C concentrations were calculated using the Friedewald equation [17].

### 2.3. DNA Analysis

Genomic DNA was prepared from white blood cells using the salting-out method [18]. Genotypes for the variant 9p21.3 were determined by polymerase chain reaction (PCR).

### 2.4. Genotyping

Allele specific primers for the ancestral (C) and derived (G) alleles of rs1333049: C > G, were designed to selectively amplify the relevant target sequences [19]. The sequence of the forward primer for the C allele was 5′-TCC TCA TAC TAA CCA TAT GAT CAA CAG TTC-3′ and for G allele the sequence was 5′-TCC TCA TAC TAA CCA TAT GAT CAA CAG TTG-3′. The internal control primer sequence was 5′-GAA GAT CAT ACC CGA AGT AGA GCT GC-3′. For all the forward primers a common reverse primer was used, with the sequence 5′-ATA CCA CAG TGA ACA TAA TTG TGC ATA CAT-3′. Amplification of ancestral and derived alleles was performed separately in a total reaction volume of 20 *μ*L each, containing 0.2 *μ*M deoxynucleotide triphosphates, 1x Taq buffer, 3 mM MgCl_2_, 0.2 Mm allele specific forward primer, 0.3 *μ*M reverse primer, 0.1 *μ*M internal control primer, and 1.0 U Taq polymerase. Thermocycling consisted of an initial denaturation step of 94°C for 4 min followed by 35 cycles of 94°C for 1 mn s, 61°C for 30 s, and 72°C for 40 s and a final extension cycle of 72°C for 7 min. The amplified products consisting of a 280 bp fragment for the allele specific primers and 500 bp for the internal control were electrophoretically separated on 2% agarose gels, and DNA bands were visualized by UV transillumination, the image was documented using the BioCap MW software (ver. 11.01, Vilber Lourmat, France) and the genotype data were then calculated.

### 2.5. Statistical Analyses

All statistical analysis was performed using version 11.0 of the Statistical Package for the Social Sciences: SPSS (SPSS Inc., Chicago, Illinois, USA). Continuous variables are presented as mean ± standard deviation, and categorical data are summarized as frequencies or percentages. Normal distribution of continuous variables was evaluated with the Kolmogorov-Smirnov test, and differences among groups were analyzed by one-way analysis of variance (ANOVA). For categorical variables, differences between groups were evaluated by the chi-square test. Odds ratios (ORs) of CAD for CC genotype on rs1333049, and other risk factors, were estimated by multivariate logistic regression analyses. The significance of multiplicative interactions between CC genotype and covariates was determined by a logistic regression model. The relationship between genotype and coronary disease was evaluated with the Mann-Whitney *U* test. A 2-sided probability level of ≤0.05 was considered significant.

## 3. Results

### 3.1. Clinical Characteristics and Genotype Frequencies

Table 1 shows clinical features and genotype frequencies of control group, diabetic patients with normal arteries, CAD patients with Gensini score <40, and CAD patients with Gensini score >40. Compared to the diabetic population, patients of the control CAD group are younger and have a decreased percentage of family history of CAD (all *p* < 0.05). As shown, patients with no stenosis (Group 1) are younger than patients with CAD (Groups 2 and 3), all *p* < 0.05. There is a significant linear increase in the percentages of subjects with a family history for CAD and in cholesterol, TG, and LDL levels (all *p* < 0.05). No significant differences in sex, HbA1c, and HDL-C levels were found among the subgroups (all *p* > 0.05). The percentages of patients treated with insulin were significantly correlated with the increasing extent of the coronary atherosclerosis. There is a significant linear increase in the percentages of subjects with hyperlipidemia, obesity, and smoking history and in BMI values. The homozygous CC genotype was significantly more common in patients with both CAD and T2D groups (*p* < 0.05).

### 3.2. Association between rs1333049 Polymorphism, Biochemical Measurements, and Clinical Features

Biochemical measurements and clinical features with respect to various genotypes are listed in Table 2. Subjects with family history of CAD, hypertension, obesity, smoking, insulin, and hyperlipidemia percentages were significantly higher in CC genotype carriers (all *p* < 0.05), likewise for BMI, LDL-C, and TG values. There were no significant differences in changes of gender, cholesterol, HDL-C, and HbA1c value among the three genotypes in the whole population (all *p* > 0.05) (Table 2).

### 3.3. Association between rs1333049 Polymorphism Severity and Early-Onset of CAD

In Table 3 there was an association between CC genotype and severity of CAD in all patients (*p* < 0.001), since Gensini scores differ significantly between CC genotype carriers and non-CC genotype carriers (49 ± 21,3 versus 14,87 ± 25,22). This study also showed that diabetic CC genotype carriers with early-onset CAD had significantly higher Gensini scores than diabetic non-CC genotype carriers (50,98 ± 18,71 versus 11,34 ± 22,80; *p* < 0.001).

### 3.4. Multivariable Analysis

A multiple logistic regression analysis was performed with CAD severity (Gensini score < or >40) as the dependent variable and the following as risk factors: age, family history of CAD, hypertension, smoking, hyperlipidemia, obesity, BMI, triglycerides, LDL-C, and insulin (Table 4). Analysis showed that BMI (OR: 1.03; 95% CI: 0.92–1.12; *p* = 0.03), TG levels (OR: 2.4; 95% CI: 1.82–3.29; *p* < 0.001), LDL-C levels (OR: 1.76; 95% CI: 1.42–2.18; *p* < 0.001), family history of CAD (OR: 1.85; 95% CI: 1.01–3.89; *p* = 0.01), hypertension (OR: 3.26; 95% CI: 1.52–6.98; *p* < 0.001), obesity (OR: 1.53; 95% CI: 1.19–2.01; *p* = 0.02), smoking (OR: 1.8; 95% CI: 1.2–3.89; *p* < 0.001), insulin (OR: 4.71; 95% CI: 2.3–9.65; *p* < 0.001), and hyperlipidemia (OR: 2.56; 95% CI: 1.12–4.06; *p* < 0.001) are important risk factors of CAD severity in type 2 diabetic patients. Further analysis showed that CC genotype was associated with CAD in Group 3 patients (OR 5.77; 95% CI 3.29–10.12; *p* < 0.001) and Group 2 patients (OR 1.36; 95% CI 1.06–1.52; *p* = 0.02) with increasing severity of CAD compared with Group 1 patients without CAD (OR 0.18; 95% CI 0.1–0.31; *p* = 0.2) and with the control CAD group (OR0.22; 95%CI 0.1–0.43; *p* = 0.21). So, CC genotype of rs1333049 increases the severity of CAD in Tunisian type 2 diabetic patients.

## 4. Discussion

The present study has examined the CAD risk factors changes according to CAD severity and the association of CC genotype of rs1333049 with the severity and early-onset of CAD among type 2 diabetic subjects. The principal finding of this study is that the severity and early-onset of CAD are related to the CC genotype of rs1333049.

Many genome-wide association studies have shown that genetic variations on chromosome 9p21.3 were associated with increased risk of diabetes and CAD in the general population [1–3, 8].

Interestingly, Doria et al. [20] observed that the homozygous GG genotype of rs2383206 on chromosome 9p21.3 had a greater effect on the risk of CAD in diabetic patients than CC genotype in the general population. In the present study, we found that the homozygous CC genotype of rs1333049 was associated with severity of CAD in diabetic patients. Furthermore, in our population, CC genotype carriers had significantly higher smoking, hypertension, hyperlipidemia, family history of CAD, and obesity percentages than non-CC genotype carriers; it is the same for BMI, LDL-C, and TG levels. These observations suggest that these factors are important risk factors of CAD for the diabetic patients in the Tunisian population.

The mechanisms of severity of CAD and T2D in patients with acute coronary syndrome (ACS) are complex and not so clear at present. While many genetic studies [21], for CAD and T2D, have identified the 9p21.3 locus as a common risk region, there are no functional variants (noncoding variants) within this region. Therefore, the relation between the 9p21.3 locus and the genetic principles of these two diseases remains poorly understood.

The essential mechanisms by which SNPs on chromosome 9p21.3 contribute to early-onset and severity of CAD in diabetes remain unclear.

In a family-based study, Meng et al. [16] observed that chromosome 9p21.3 is associated with early-onset CAD in the Irish population. In the present large-sample study, we found that after investigating their association with traditional risk factors homozygous CC genotype carriers were found to have an increased risk for early-onset CAD in diabetic patients. Moreover, among diabetic patients with early-onset CAD, CC genotype carriers had significantly higher Gensini scores than non-CC genotype carriers. These observations suggest that there may be a common genetic basis for diabetes and CAD in a homozygous genotype of chromosome 9p21.3 [5].

## 5. Conclusion

This study demonstrated that 9p21.3 polymorphism is significantly associated with CAD in a Tunisian population, and the homozygous CC genotype of rs1333049 confers a magnified risk of early-onset and severe CAD in type 2 diabetic patients.

## Figures and Tables

**Table 1:** Baseline clinical characteristics and biochemical assessments.

	Control CAD group	Total of the diabetic population	Group 1	Group 2	Group 3	*p*
*N*	101	273	138	36	100	—
Gender: male/female (%)	56,4/43,6	52,7/47,3	52,2/47,8	58,3/41,7	52/48	0,651
Age (years)	56,27 ± 16,75	62,82 ± 12,26	60,36 ± 13,42	65,92 ± 9,17	65,26 ± 10,91	0,003
Family history for CAD (%)	46,9	60,4	50,7	63,9	72	0,007
Hypertension (%)	40,1	59,3	47,8	63,9	75	<0,001
BMI	27,21 ± 3,99	29,59 ± 4,51	28,63 ± 4,74	30,03 ± 2,02	30,73 ± 4,55	0,002
Obesity (%)	30,4	47,3	35,5	61,1	58	0,001
HbA1c (mmol/L)	7,1 ± 3,52	10,52 ± 4,20	10,27 ± 3,94	10,08 ± 4,30	11,06 ± 4,50	0,251
Smoking (%)	31,9	42,1	33,3	58,3	49	0,006
Cholesterol (mmol/L)	4,58 ± 1,3	4,72 ± 1,12	4,73 ± 1,14	4,52 ± 0,87	4,78 ± 1,18	0,414
LDL (mmol/L)	2,91 ± 1,13	3,73 ± 1,45	3,28 ± 1,22	3,86 ± 1,35	4,29 ± 1,57	<0,001
HDL (mmol/L)	1,14 ± 0,44	1,23 ± 0,52	1,26 ± 0,51	1,17 ± 0,40	1,22 ± 0,58	0,455
TG (mmol/L)	1,27 ± 0,64	2,21 ± 1,29	1,76 ± 1,06	2,07 ± 0,98	2,91 ± 1,40	<0,001
Hyperlipidemia (%)	55,6	49,5	—	97,2	100	<0,001
Insulin (%)	—	52,4	36,2	61,1	72	<0,001
Rs1333049 genotypes						
GG (%)	72,3	25,3	40,6	11,1	10	0,377
GC (%)	22,8	46,2	52,9	47,2	36	0,136
CC (%)	5	28,6	6,5	41,7	54	<0,001

Data are number (%) and mean ± SD. BMI: body mass index; CAD: coronary artery disease; HbA1c: glycosylated hemoglobin; HDL-C: HDL cholesterol; LDL-C: LDL cholesterol; TC: cholesterol total; TG: triglycerides.

**Table 2:** Changes in biochemical measurements and clinical characteristics among the three genotypes in the three groups of the diabetic population.

	Rs1333049			
	GG (*n* = 69)	GC (*n* = 126)	CC (*n* = 78)	*p*
Gender: male/female (%)	31/38	54,8/45,2	56,4/43,6	0,314
Age (years)	62,04 ± 13,66	62,22 ± 12,91	64,46 ± 9,59	0,037
Family history for CAD (%)	56,5	50	80,8	<0,001
Hypertension (%)	52,2	59,5	65,4	0,026
BMI	28,24 ± 4,23	29,24 ± 4,73	31,34 ± 3,84	<0,001
Obesity (%)	30,4	40,5	73,1	<0,001
HbA1c (mmol/L)	10,24 ± 3,73	10,32 ± 4,23	11,08 ± 4,53	0,379
Smoking (%)	36,2	38,1	53,8	0,045
Cholesterol (mmol/L)	4,89 ± 1,17	4,56 ± 1,19	4,83 ± 0,93	0,081
LDL (mmol/L)	3,53 ± 1,34	3,73 ± 1,48	3,91 ± 1,49	0,028
HDL (mmol/L)	1,37 ± 0,65	1,19 ± 0,48	1,19 ± 0,44	0,06
TG (mmol/L)	1,87 ± 1	2,13 ± 1,21	2,66 ± 1,52	0,001
Hyperlipidemia (%)	18,8	42,1	88,5	<0,001
Insulin (%)	44,9	47,6	66,7	0,011

Data are number (%) and mean ± SD. BMI: body mass index; CAD: coronary artery disease; HbA1c: glycosylated hemoglobin; HDL-C: HDL cholesterol; LDL-C: LDL cholesterol; TC: cholesterol total; TG: triglycerides.

**Table 3:** Association of rs1333049 CC genotype with severity and early-onset of CAD in diabetic patients.

	Rs1333049		
	CC (*n* = 78)	Non-CC (*n* = 237)	*p*
Gensini score			
All patients	49 ± 21,3	14,87 ± 25,22	<0,001
Early-onset CAD	50,98 ± 18,71	11,34 ± 22,80	<0,001
Late-onset CAD	45,57 ± 25,26	18 ± 26,95	<0,001

Data are mean ± SD. CAD: coronary artery disease.

**Table 4:** Multivariable logistic regression analysis of independent determinants for CAD.

	Control CAD group			Diabetic population								
				Group 1			Group 2			Group 3		
	OR	95% CI	*p*	OR	95% CI	*p*	OR	95% CI	*p*	OR	95% CI	*p*
rs1333049 (CC versus non-CC)	0,22	0,1–0,43	0,21	0,18	0,1–0,31	0,2	1,36	1,06–1,52	0,02	5,77	3,29–10,12	<0,001
Family history for CAD (%)	0,35	0,11–0,69	0,12	0,56	0,27–1,16	0,12	2,05	1,09–3,87	0,02	1,85	1,01–3,89	0,01
Hypertension (%)	0,67	0,22–0,98	0,24	0,31	0,17–0,57	0,52	1,28	1,14–1,56	0,002	3,26	1,52–6,98	<0,001
BMI	0,80	0,60–1,07	0,14	0,97	0,88–1,07	0,58	1,02	0,94–1,13	0,04	1,03	0,92–1,12	0,03
Obesity (%)	0,96	0,56–1,34	0,09	1,54	0,58–4,12	0,38	1,04	0,44–2,46	0,04	1,53	1,19–2,01	0,02
Smoking (%)	0,42	0,13–0,77	0,99	0,52	0,25–1,09	0,08	2,11	1,12–3,99	0,02	1,8	1,2–3,89	<0,001
LDL (mmol/L)	0,48	0,12–0,83	0,28	0,56	0,45–0,71	0,04	1,64	1,5–1,82	<0,001	1,76	1,42–2,18	<0,001
TG (mmol/L)	0,21	0,12–0,33	0,88	0,4	0,3–0,54	0,07	1,45	1,33–1,61	<0,001	2,4	1,82–3,29	<0,001
Hyperlipidemia (%)	0,76	0,22–0,95	0,08	—	—	—	1,37	1,01–1,67	0,01	2,56	1,12–4,06	<0,001
Insulin (%)	—	—	—	0,21	0,1–0,43	0,9	4,3	2,34–7,87	<0,001	4,71	2,3–9,65	<0,001

BMI: body mass index; CAD: coronary artery disease; LDL-C: LDL cholesterol; TG: triglycerides.

## Abbreviations

ACS: Acute coronary syndrome
ANOVA: Analysis of variance
BMI: Body mass index
CAD: Coronary artery disease
DNA: Deoxyribonucleic acid
HbA1c: Glycosylated hemoglobin A1c
HDL-C: HDL cholesterol
LDL-C: LDL cholesterol
OR: Odds ratio
PCR: Polymerase chain reaction
SNP: Single nucleotide polymorphism
SPSS: Statistical Package for the Social Sciences
TC: Total cholesterol
TG: Triglyceride
T2D: Type 2 diabetes.
